# Short loop functional commonality identified in leukaemia proteome highlights crucial protein sub-networks

**DOI:** 10.1093/nargab/lqab010

**Published:** 2021-03-01

**Authors:** Sun Sook Chung, Joseph C F Ng, Anna Laddach, N Shaun B Thomas, Franca Fraternali

**Affiliations:** Department of Haematological Medicine, King’s College London, London, SE5 9NU, UK; Randall Centre for Cell and Molecular Biophysics, King’s College London, London, SE1 1UL, UK; Randall Centre for Cell and Molecular Biophysics, King’s College London, London, SE1 1UL, UK; Randall Centre for Cell and Molecular Biophysics, King’s College London, London, SE1 1UL, UK; Department of Haematological Medicine, King’s College London, London, SE5 9NU, UK; Randall Centre for Cell and Molecular Biophysics, King’s College London, London, SE1 1UL, UK

## Abstract

Direct drug targeting of mutated proteins in cancer is not always possible and efficacy can be nullified by compensating protein–protein interactions (PPIs). Here, we establish an *in silico* pipeline to identify specific PPI sub-networks containing mutated proteins as potential targets, which we apply to mutation data of four different leukaemias. Our method is based on extracting cyclic interactions of a small number of proteins topologically and functionally linked in the Protein–Protein Interaction Network (PPIN), which we call short loop network motifs (SLM). We uncover a new property of PPINs named ‘short loop commonality’ to measure indirect PPIs occurring via common SLM interactions. This detects ‘modules’ of PPI networks enriched with annotated biological functions of proteins containing mutation hotspots, exemplified by FLT3 and other receptor tyrosine kinase proteins. We further identify functional dependency or mutual exclusivity of short loop commonality pairs in large-scale cellular CRISPR–Cas9 knockout screening data. Our pipeline provides a new strategy for identifying new therapeutic targets for drug discovery.

## INTRODUCTION

The use of reliably assembled Protein–Protein Interaction Networks (PPINs) has become common practice in the last two decades in the quest to identify biological pathways and cellular mechanisms related to newly discovered genes or disease related proteins (1–3). In recent years, the quality and quantity of interactions shown to occur experimentally has increased substantially, particularly due to large-scale studies using yeast-two-hybrid (4) and a panel of different protein purification/mass spectrometry methods (5–9). Additionally, an increasing number of public protein interaction data sources (10–12) and a collaborative effort through the International Molecular Exchange (IMEx) consortium (13) are now in place to improve coverage, quality and integrity of proteome data. However, accurate and comprehensive compilation of such heterogeneous databases is a challenging task and the currently available information is still incomplete. Therefore, we are far from having a complete proteome map for any human cell type.

Concurrently with progress in the field of PPINs, whole genome and exome sequencing projects have identified disease-related mutations and population-related variants in protein-coding regions (14,15). The former, disease-related mutation information, includes data from cell lines and samples from patients, and is collated in the OMIM (16), COSMIC (17), TCGA (18) and ClinVar (19) databases. The latter, population-related variation information, is collected in dbSNP (20), 1000 Genomes (21), ExAC and gnomAD (22). These shared resources have enabled the discovery of disease associations of mutations in the human genome, a subset of which affect protein sequences (23,24). In establishing the impact of these mutations on protein stability and function in the cell, one possibility is to evaluate the impact of disease-causing mutations on the protein 3D structure, when this is available. The 3D structure of a protein is more conserved during evolution than its linear sequence (14); therefore, these evaluations have been used as a better proxy to predict the impacts of mutations on the biological function(s) of the mutated protein (25–27). Unfortunately, structural determination is still challenging and therefore not available for all proteins and their interactors, making a large-scale protein structure analysis of cellular PPINs unfeasible. As a consequence, the structure–sequence gap is still large (28) and even the use of homologous sequence(s) cannot compensate for such missing information. Therefore, understanding the effects of many genetic variants and mutations on biological functions and the interplay among these in curated PPINs is still very challenging. Specifically, the prediction of these mutual effects is an important challenge, as it has been suggested that many complex traits are driven by large numbers of mutations, each of which has a potentially small effect on cellular function, which is propagated through a PPIN to affect biologically important core functions (29).

Different approaches have been developed to analyse such biological ‘Big Data’ effectively (30–32) and graph theory based approaches have improved our understanding of large-scale data networks in general, and PPINs (33–35) in particular. In this case, proteins are nodes and their interactions are edges. Various PPIN-wide (global) and PPI (local) network properties have been suggested to measure connectivity of the network and to identify sub-network modules (36,37). Previously, we defined short-loop network motifs, cyclic interactions of a small number of proteins, as an intrinsic feature of PPINs topologically and biologically (38). We demonstrated that these loops not only contribute to define the wiring and topological properties of the network, but also have a critical role in performing dedicated biological functions. It was highlighted that short loop network motif profiling is advantageous in assessing the quality of the network and is useful in extracting biologically functional sub-networks.

In the study presented here, we explore the effects of genetic mutations on PPINs of four major Blood Cancers that affect the production of mature white blood cells of myeloid and lymphoid lineages that are necessary for normal immune responses (acute myeloid leukaemia (AML), acute lymphocytic leukaemia (ALL), chronic lymphocytic leukaemia (CLL) and chronic myeloid leukaemia (CML)) and investigate AML more closely. Acute myeloid leukaemia is heterogeneous, and patients are sub-classified according to cell morphology and genetic abnormalities by the French–American–British (39) and World Health Organization working group (40) criteria. AML was the first cancer whole genome sequenced (41) and to date several thousand mutations have been identified in patients with AML, some of which are driver mutations that have been used as genomic identifiers of patient sub-groups (42,43). Recent single-cell RNA sequencing studies have shown that in individual AML patients, the blood cancer is comprised of abnormal cell clones, each clone with a different combination of mutations, and selection and expansion of different abnormal cell clones occurs during disease progression (44–46). In spite of our knowledge of gene mutations in AML, identifying key proteins for targeted drug therapies presents a considerable challenge and currently there are no effective drug treatments for AML. Furthermore, the combined effects of the mutated proteins present in this Blood Cancer, rather than the effects of individual mutations, have not been identified. Drugs targeting a few mutated proteins, including FLT3 and IDH1/2 have been tested in clinical trials and trials of drugs targeting several other mutated proteins are in progress (47–49). However there are reports of patient relapses in trials of FLT3 and IDH inhibitors for reasons including gene mutations and changes in gene expression that overcome the effects of the drugs (47).

Our current study seeks to pinpoint functionally important protein ‘modules’ containing proteins mutated in AML to identify proteins other than the mutated protein that could be drug targets (47). To clarify some of the underlying phenomena in Blood Cancer, we generated a unified large-scale human PPIN (UniPPIN) from multiple reliable sources. Mutations in leukaemias were mapped to the UniPPIN and our short-loop network motif profiling method was applied to extract leukaemia, cancer and non-disease related mutation sub-networks. The ratio of short loops and the functional consensus across sub-networks was compared to infer features of each network and biological functions enriched in the short loops of different leukaemias. Furthermore, we propose a novel module-based concept to compare indirectly connected proteins that share protein interactions that we named ‘short loop commonality’. This has enabled us to use proteins-of-interest as ‘seeds’ to identify neighbours in the network that represent functionally important protein modules. In addition, their mutual functions have been inferred by analysing CRISPR–Cas9 gene-dependency screening data. We show here that using the short loop profiling method, combined with information on pathogenic mutations, identifies enriched co-functional units and intimate protein interaction components with hotspot or driver mutations that could be exploited effectively for drug target screening.

## MATERIALS AND METHODS

### Protein–protein interaction datasets

PPIs are represented by graph models consisting of nodes of proteins and edges of their interactions. We integrated a data set of 10 different human protein–protein interaction resources including collated databases (10–12,50,51) and recent large-scale studies identifying PPIs. The studies include a broad binary proteome map by screening pairwise combinations of over 10 000 human open reading frames (52) with yeast-two-hybrid assays (4), collated published evidence (String) (12), affinity purification/mass spectrometry-based networks using different ‘bait’ proteins (green fluorescent protein-tagged (GFP) for (6) and FLAG-HA epitope tags for the BioPlex network (5)) and co-fraction/mass spectrometry-based networks (7,53). Protein interaction information from all datasets except for String (12) is derived from direct experimental evidence from the laboratory concerned and only high confidence scored interaction information (above 0.5) is counted from the String database (12). Those recent studies and the stringent criteria applied supported the reliability of the UniPPIN dataset; however, it could still include false positive information due to limitations in current experimental procedures (54). The UniProt Accession number (55) was used to amalgamate different formats of each dataset and duplicate interactions and self-loop interactions are removed in the UniPPIN. The details of the resources are described in Supplementary Table S1 and the data are available at https://github.com/suns-chung/ShortLoopCommonality.

### Resources of human genetic variations or single-nucleotide polymorphisms and mutations in cancer

The Catalogue Of Somatic Mutations In Cancer (COSMIC) (17), the largest cancer mutation database deposited from numerous research institutes worldwide, was used to download cancer and leukaemia related variation information (v80 (Feb 2017)). The database contains information on the somatic mutations present in samples isolated from individual cancer patients. The methods used include whole genome sequencing, exon sequencing, targeted exon/codon sequencing and specific single nucleotide change analyses. Protein mutations reported for several sub-types of four common leukaemias were retrieved: acute myeloid leukaemia (AML), chronic myeloid leukaemia (CML), acute lymphoid leukaemia (ALL) and chronic lymphoid leukaemia (CLL). These four were chosen as they affect different haematopoietic white cell lineages, namely myeloid and T- and B-lymphoid cells. The histology terms and their classification are listed in Supplementary Table S2. Mutation types were selected which result in amino acid changes: substitution nonsense, substitution missense, insertion inframe, insertion frameshift, deletion inframe, deletion frameshift and complex or compound mutation. ‘Whole gene deletion’ and ‘nonstop extension’ mutations are included but both mutations were rarely observed (56 out of 69 334). In addition, only genes with these non-synonymous mutations in at least two different patients were included. The datasets with ENSEMBL Gene identifiers (56) were mapped into the UniProt Accession number (55) for the analyses in this project. The full lists of frequently mutated proteins in each leukaemia are available in Supplementary Table S3a–d.

The leukaemia related mutation datasets were compared with somatic cancer mutation dataset or non-disease human genetic variation information collected from COSMIC and dbSNP of which mutation types are point mutations or single-nucleotide variants (SNVs) giving rise to non-synonymous mutations.

We collected data from different public resources: for disease-associated variant information, non-synonymous SNVs from COSMIC (17); for non-disease related information, a subset of dbSNP (20) grouped as common mutations. The details of each dataset and the criteria are: (i) COSMIC exonic variants in variant call format (VCF) (CosmicCodingMuts.vcf) downloaded (v80, February 2017) and (ii) ‘common’ variants from dbSNP defined in the National Center for Biotechnology Information, U.S. National Library of Medicine (NCBI) database, ‘germline origin and a minor allele frequency (MAF) ≥ 0.01 in at least one major population, with at least two unrelated individuals having the minor allele’ (57).

These variant datasets in variant call format (VCF) were mapped to the ENSEMBL protein sequences (GRCh37) (56) by using the Variant Effect Predictor (VEP) software tool (58). The datasets were further filtered for missense variants which map to canonical protein sequences. For each protein, the frequency of localized variants was normalized by the length of amino acid sequences in the protein defined as

$$\begin{equation*}{\rm{Frequency\ of\ \ }}\left( {{\rm{nsSN}}{{\rm{V}}_{{\rm{normalised}}}}} \right) = {\rm{\ }}\frac{{{\rm{Number\ of\ nsSNV}}}}{{{\rm{length\ of\ protein}}}}\end{equation*}$$

based on the assumption that the mutability of a protein is primarily associated with its size and that differences in amino acid composition between proteins do not have an impact on their overall mutability (nsSNV: non-synonymous single-nucleotide variants).

### Sub-networks of protein–protein interactions related to gene mutations

Proteins of each variant dataset based on the UniProt Accession number (collected on 15 March 2017) were mapped to the UniPPIN. Each dataset of mutated proteins was mapped to the UniPPIN and then mapped proteins and interactions between those proteins were extracted to construct sub-networks related to specific leukaemias or variant datasets. As an example of the labelling used, ‘AML-related protein–protein interaction network’ stands for protein interactions among proteins mutated in at least two AML patients. However, the mutations do not necessarily occur in the same patient.

### Short loop network motif profiling

The short loop network profiling approach (38) was used to analyse PPIN sub-networks containing mutations. The numbers of short loops in each network were calculated and the results were compared with randomized models. The numbers of short loops in the variant specific PPINs and randomly generated PPIN models were evaluated by statistical tests. The random sampling was conducted by selecting proteins randomly and extracting their interactions from the UniPPIN (*n* = 2000 samplings for short loops and 10 000 simulations for short loop commonality analysis). Functional analyses using GO terms (59) were carried out by measuring functional consensus, that is, the percentage of GO terms shared by proteins in a short loop as previously described (38). In addition, g:Profiler (60) and ClueGO (61) were used to measure function enrichment of proteins in different sets. The methods can measure statistical significance of given datasets (*P*-value ≤ 0.05) compared with their functional term databases. As there was no significant difference in the topology and ontology of proteins in short loops of different lengths when we applied rigorous graph dynamics and functional enrichment analyses throughout short loop lengths 3–6 for the previously studied larger network (38), only short loop interactions with length 3 were used in this study.

### Gene dependency analysis

#### Data source

CRISPR screen data from the depmap (Cancer Dependency Map, https://depmap.org) project were taken for analysis (version 19Q4). These screens measure the viability of cell lines following the knockout each gene, one by one, using a large CRISPR guide RNA library (62). These measurements had been pre-processed using CERES (63), then shifted and scaled per cell line so that each gene in each cell line is represented with one score by depmap. The median score representing a non-essential (knockout of which has no effect) gene is 0, and the median essential knockout effect is –1. These normalized scores were directly downloaded for this analysis. Only cell lines marked ‘HAEMATOPOIETIC_AND_LYMPHOID_TISSUE’ were considered, totalling to 67 cell lines. Sequence and Copy Number variant data listed on depmap (version 2019Q4, identical to that in the Cancer Cell Line Encyclopaedia [CCLE] (64)) were downloaded and mapped to the CRISPR screen data.

#### Mapping gene dependency data to short loop commonality pair

For each short loop commonality pair of proteins found in each of the leukaemia networks (AML/ALL/CML/CLL), this set of protein pairs (‘disease set’), totalling 259, were looked up in the CRISPR knockout data. We also compiled a ‘control set’ (totalling 560 protein pairs), made up of protein pairs where only one of the proteins (here denoted A) overlapped with the ‘disease set’. The other member of the pair instead shares short-loop commonality with A, but could be found only in the general UniPPIN without filtering for leukaemia-specific-mutated proteins.

For each protein pair in both the ‘disease set’ and the ‘control set’, the normalised gene dependency scores (see above) were categorized such that cell lines with scores < –1 for a given gene are labelled to be ‘dependent’ on the gene (65). To investigate the relationship between the dependency profile of two proteins (hereafter named X and Y), a two-way contingency table is then considered. Both co-dependency (i.e. cell lines found to be dependent on both X and Y) and mutual exclusivity (i.e. cell lines found to be dependent on either X or Y) were evaluated, using a Fisher’s exact test (R function fisher.test). Note that since gene dependency is typically very sparse (i.e. for a lot of genes, the cell lines are not dependent on any of them), and that certain essential genes tend to have universal dependency across all the examined cell lines, we avoided prioritizing gene pairs using solely the resulting *P*-values from this independence; instead we considered the following criteria: (i) <50% of the examined haematopoietic cell lines were dependent on each of the genes; this aims to eliminate essential, ‘housekeeping’ genes on which cell lines will depend regardless of their mutation status; (ii) >1 cell line was dependent on each of the genes in cases of mutual exclusivity. For defining co-occurrence of gene dependency, it was required to have >1 cell line where the co-occurrence was observed. With these criteria, we identified, in the ‘disease set’ 13 pairs of gene dependency mutual exclusivity and 3 pairs of co-occurrence. For the ‘control set’, we identified 4 pairs of mutual exclusivity and no cases of gene dependency co-occurrence. These data are tabulated in Supplementary Table S4.

Heatmaps were plotted to display the dependency and mutational profiles of leukaemia cell lines for a given short-loop commonality pair, as well as their shared neighbours in the UniPPIN, using the R package ComplexHeatmap (66).

### Software for data processing, analysis and visualization of networks

Python (https://www.python.org/; ver. 3.6.0) scripts were used for large-scale data processing such as UniPPIN establishment, sub-network analysis and short loop network motif profiling and they are available at https://github.com/suns-chung/ShortLoopCommonality. Gene dependency analysis and plotting were implemented in R (https://www.r-project.org/). PPINs were visualized by Cytoscape 3.6.0 (67).

## RESULTS

### Protein mutations in leukaemias reported in the COSMIC database

Mutations in leukaemias were extracted from the COSMIC database, the most comprehensive public resource for cancer mutation (68), and mapped to the UniPPIN and the potential impact on the cellular functions affected was determined as described below. Four major leukaemias, acute myeloid leukaemia (AML), chronic myeloid leukaemia (CML), acute lymphoblastic leukaemia (ALL) and chronic lymphocytic leukaemia (CLL) were chosen as they affect different haemopoietic cell lineages, lymphoid and myeloid. The pathological mechanism of CML is generally understood as it is dependent on one dominant gene fusion, *BCR-ABL* (69), whereas each of the other leukaemias are more complex as their mutations occur in many different genes. Data were retrieved from the COSMIC database after filtering to remove synonymous mutations and single-case observations. Mutations occurring in patients diagnosed with one of the four leukaemias were first analysed together to extend potential associated disease information and increase predictions of the cellular ontologies affected.

In the COSMIC database, mutation data for 32 330 leukaemia patients are available: 26 127 for AML, 2706 for CML, 1514 for ALL and 1983 for CLL. For each leukaemia, there are different numbers of mutated genes encoding proteins observed in at least two patients: 4141 proteins in AML, 318 proteins in CML, 1065 proteins in ALL and 1802 proteins in CLL (Table 2 the first column and Supplementary Tables S3 and S5). By comparing the proteins with mutations in each dataset, there are only 46 proteins (0.8%) (based on the UniProt Accession number) encoded by 42 unique genes that have mutations in all four leukaemias of which half have been annotated as oncogenes or tumour suppressor genes in the Cancer Gene Census (70) (Table 1). 39 out of 42 genes encode a single protein form but multiple isoforms were found for *TP53*, *TTN* (2 isoforms each) and *SYNE1* genes (3 isoforms). Also, proteins encoded by 27 out of 42 genes are involved in processes highlighted as the ‘hallmarks of cancer’ (71,72) (Table 1 and Supplementary Table S6). Although the functions of specific proteins have been discovered, for example in the Jak–STAT pathway (73), the way that mutations in combinations of these proteins affect the PPIN and cellular processes affected has yet to be defined.

**Table 1.** Proteins mutated in common in AML, CML, ALL and CLL and their functions in cancer. The mutated proteins in each of the four leukaemias, based on the UniProt Accession number are listed with their gene names. They were compared with the Cosmic cancer gene census information (70) (Acc: Accession number, TSG: tumour suppressor gene) and the processes related to the hallmarks of cancer (71,72) (the gene annotations are assigned as described in (106): shaded green). 32 out of 42 genes have at least one of the cancer-associated annotations listed in this table and the whole list is in Supplementary Table S6

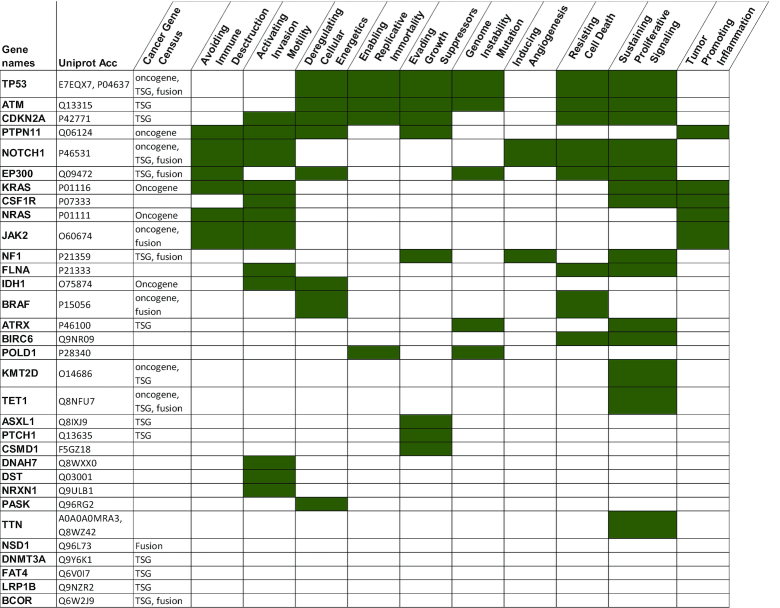

### Comparison of sub-networks by short loop network motif profiling

To investigate how proteins mutated in leukaemia may affect the functions of other proteins and hence the associated pathways, we identified their interacting protein partners in the human PPIN. In order to capture information on as many PPIs as possible, multiple resources of protein interaction information involving comprehensive databases and large-scale studies were employed to establish a Unified PPIN (UniPPIN), as described in ‘Materials and Methods’ section, containing 19 370 proteins with 385 879 interactions. Protein mutations in all cancers including different leukaemias were collected from the COSMIC database (17) and non-disease related non-synonymous single-nucleotide variants (nsSNVs) from several databases as described in ‘Materials and Methods’. The non-disease variations were obtained from a subset of dbSNP labelled as ‘common’ for variants without known pathogenic relevance, specifically of germline origin and a minor allele frequency (MAF) ≥ 0.01 in at least one of the 1000 Genomes Population as defined in (20,57). All the proteins mutated in each leukaemia were mapped to the UniPPIN. Although the UniPPIN is a large-scale collection, there are gaps and more than one third of the proteins mutated in leukaemias do not map to the UniPPIN (Table 2). This is particularly true for human membrane proteins, for which protein interaction data are sparse (14) and information we have is usually based on biological studies of individual receptors. However, the leukaemia-specific PPINs involve between half and two-thirds (53–66%) of mutated proteins in each leukaemia (Table 2).

**Table 2.** Short loop network motif (length = 3) profiling for each mutation dataset. The number of short loops (length = 3) was counted and assigned with proteins having mutations in the four different leukaemias, two of which affect myeloid and two affect lymphoid cells. ‘Common mutation’ or non-pathogenic mutation is obtained from dbSNP and filtered based on the definition in the NCBI database, ‘germline origin and a minor allele frequency (MAF) ≥ 0.01 in at least one major population’. In each case, the mutated protein was mapped onto UniPPIN. Other network properties are also shown, such as the number of proteins, the number of proteins in the UniPPIN with ratios from the original number of proteins in parentheses and PPIs in the specific PPINs. In addition, the last column shows the functional consensus of the short loops, measured as the percentage of short loops having shared functions among proteins in the same loop (FC: Functional consensus)

	Number of proteins	Number of proteins in UniPPIN	Number of PPIs	Number of Loop3	Ratio Loop3/PPIs	Loop3 FC (%)
**AML**	4 141	2 609 (63%)	14 119	17 443	1.24	88.17
**CML**	318	169 (53%)	367	228	0.62	100.00
**ALL**	1 065	667 (63%)	2 256	1 532	0.68	96.21
**CLL**	1 802	1 189 (66%)	4 364	3 088	0.71	97.77
**COSMIC**	18 459	15 759 (85%)	336 216	1 816 503	5.40	91.38
**Common mutation (non-pathogenic from dbSNP)**	17 065	14 214 (83%)	233 470	714 033	3.06	87.68
**UniPPIN**	19 370	19 370	385 879	2 085 705	5.41	90.19

In order to extract information from this large network on the local interactions of mutated proteins and the cellular functions affected by such mutations, we used the Short Loop profiling method computing cyclic interactions of a small number of proteins (38) to investigate sub-networks of proteins in the UniPPIN mutated in each of the four leukaemias. The datasets were compared by two quantitative analysis steps: (i) counting the number of short loops (length = 3) that are present in each dataset and (2) measuring the consensus of the functions of proteins in each of the short loops. We also analysed short loops of length = 4 but found no significant differences with short loops of length = 3 (data not shown).

The number of short loops correlates with the number of proteins and PPIs in a network (Pearson correlation score = 0.96 ± 0.02, *P*-value < 4E-05) and so these were normalized by the number of PPIs, as described previously (38) (Table 2). The short loops for leukaemia-specific mutations in each of the four leukaemias were analysed. We find the normalized ratio of short loops of length 3 in AML (1.24) is significantly higher than that for all other leukaemias. It is also slightly higher (*z*-score = 1.32) than the normalized ratio of randomly generated PPINs (the number of random samples = 2000, the average number of proteins in randomly sampled networks = 2602, sample mean of loop3 ratio = 0.95, sample standard deviation = 0.21, sample standard error = 0.0048) (Supplementary Figure S1). This further confirms our findings that short loops are an intrinsic topological property of PPINs (38) and they represent an additional network metric, similar to neighbourhood connectivity (74) (Supplementary Table S7). These analyses show that short loops of three proteins are particularly enriched in the AML dataset and therefore proteins mutated in AML may have more inter-connections, which suggests that they are involved in more cooperative functions. The proteins frequently found in AML short loops include ATP-dependent RNA helicases (DDX3X, DDX17, DHX15, EIF4A1), splicing factors (U2AF2, PRPF8), heterogeneous nuclear ribonucleoproteins (HNRNPU, HNRNPK, HNRNPL, HNRNPF), paraspeckle protein (PSPC1) and ribosomal proteins (RPL6, RPS11, RPS16) (Supplementary Table S8).

**Figure 1.**
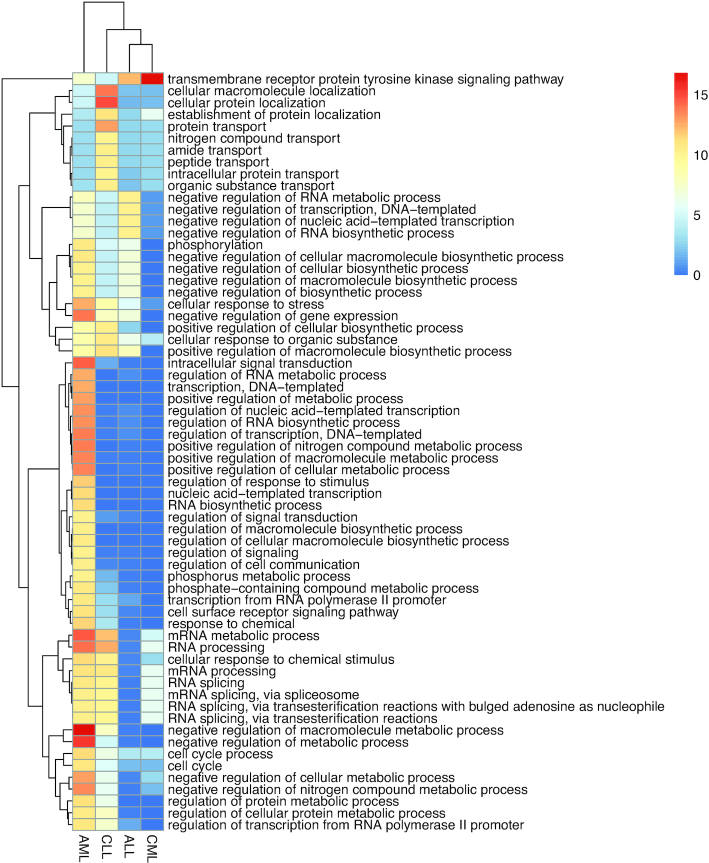
Enrichment of gene ontology in short loops of different leukaemias. Short loops in each leukaemia network were annotated using g:Profiler (60), and the relative frequency (%) of loops enriched in each gene ontology (GO) biological process was quantified. These relative frequencies (in percentage terms) were represented using a heat scale. Only GO terms represented in at least two leukaemia networks are included here. Heatmap produced using the R pheatmap package (107).

In addition, to compare collaborative functions of proteins mutated in different leukaemias, the overall functional enrichment of short loops was measured quantitatively by calculating the percentage of shared GO Biological Process terms among the short loop proteins, which we define as ‘functional consensus’ (38). This measures commonality of the functions in a loop independently of the level in the GO hierarchy and independently of the functional associations of the overall network containing the short loops. The ratio of the functional consensus in a network is calculated by the ratio of short loops having a functional consensus to all short loops ( $ = \frac{{{\rm{number\ of\ short\ loops\ with\ functional\ consensus}}}}{{{\rm{total\ number\ of\ short\ loops\ in\ a\ network}}}}{\rm{\ }} \times {\rm{\ }}100{\rm{\ }}( {\rm{\% }} )$). In the previous study, we highlighted that short loops in human PPINs consist of proteins with a high degree of functional consensus (i.e. >95% of short loops share at least one function) (Figure 5 in (38)). In particular 45–59% of the short loops in the high-confidence human PPIN (53) have a higher functional consensus ratio (>75% functional consensus). Here, we confirm these previous functional consensus analysis results (38) and find that for human UniPPIN and all subnetworks being analysed, generally ∼90% of short loops share at least one GO Biological Process term. Therefore, a short loop can be a ‘biological functional unit’ of the protein interaction network (Table 2). The biological functional units of short loop networks containing mutated proteins in the four leukaemias were then determined. The short loops of length 3 in CML, ALL and CLL have a higher ratio of functional consensus than those of the PPIN containing somatic mutations in all cancers (Table 2). On the other hand, short loops in AML and non-disease related ‘common’ variation PPINs have lower functional consensus than short loops in the UniPPIN (90.19%) and COSMIC (91.38%), indicating the enrichment of cellular functions as we discovered previously (38). The lower functional consensus of short loops in AML indicates that proteins mutated in AML play roles in a wide range of biological processes as GO classifications of the functions in AML short loops tend to be general and less specific (Figure 1 and Supplementary Table S9) including metabolic processes, signal transduction and gene expression. For example, nucleophosmin (NPM1) is mutated in 50–60% of AML patients with normal karyotype (75,76) and the interaction between NPM1 and cellular tumour antigen p53 (TP53) (77) makes short loop interactions with 25 different proteins such as cell cycle related proteins (CDKN2A, RB1), ribosomal proteins (RPL6, RPS16, RPS13), histone binding (EP300, RBBP4), actin binding (FLNA) and ubiquitin (OTUB1) (78). Therefore, the results of the short loop network motif profiling of the leukaemia-specific PPINs and the functions affected by mutations not only reflect the complexity of the diseases, but also show that mutations in AML affect a wider range of cellular functions than those affected by mutations in the other three leukaemias or in other cancers (pan-cancer analysis). Thus, such distinctness led us to focus on proteins mutated in AML and their PPIs.

### In-depth analysis of protein mutations in AML

**Figure 2.**
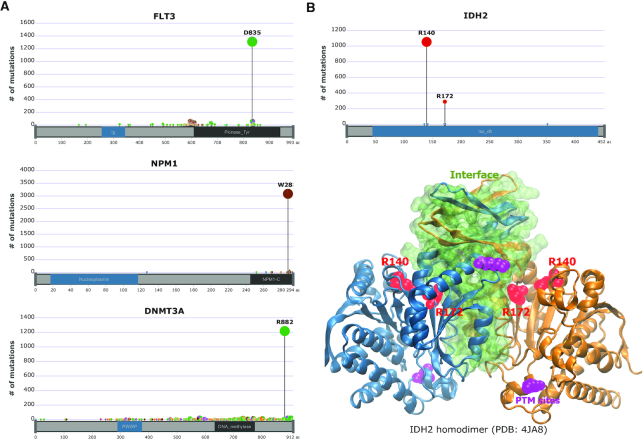
Mutation locations of frequently mutated proteins in AML patients mapped on protein domains. About 7000 proteins are mutated in AML patients according to the COSMIC (v80) database. (**A** and **B**, top) Among them, mutation information of most frequently mutated proteins, FLT3, NPM1, DNMT3A and IDH2 is visualized by g3viz tool. Each plot describes an amino acid sequence of the protein with domain information (*x*-axis) and the number of mutations on specific positions (*y*-axis). (B, bottom). On the IDH2 homodimer 3D structure (PDB: 4JA8; blue and yellow), IDH2 hotspot mutation locations, R140 and R172, are annotated in red. Post-translational modification (PTM) sites and PPI interface are also shown in purple beads and green surface, respectively.

In order to understand the implications of mutated residues at the atomistic scale and the relationships with the functional groups and active sites, we examined locations of frequently occurring mutations in AML at the amino acid sequence and 3D structural levels. Based on the COSMIC database (v80, February 2017), there are >7000 proteins that are affected by mutations observed in AML patients (including single patient occurrence) (Supplementary Table S8). This is far larger than individual studies (42,79). We pooled data of all AML patients to identify not only predominant mutation types in proteins such as FLT3 (in-frame insertion), NPM1 (frameshift insertion), CEBPA (in-frame deletion), TET2 (nonsense, in-frame deletion) and ASXL1 (frameshift, nonsense), which have been used as genomic classifiers (42), but also the enrichment of mutations in a single amino acid position or those which localize in close vicinity in the 3D protein structure, defined as ‘mutation hotspots’. Such hotspots are composed of amino acid positions with a high mutation frequency as defined in (80). In proteins frequently mutated in AML (34 proteins having mutations observed in >100 patients), more than half of these proteins (19 out of 34) have hotspot mutations (Supplementary Table S10 and examples in Figure 2). Hotspot mutations account for between 50% and 99% of the mutations in these proteins. Interestingly, these mutation hotspots are located near protein binding or interaction sites (<10 amino acid residues), when mapped on available protein 3D structures (Supplementary Table S10 and Figures 2B and 5). In the case of FLT3, the *FLT3-ITD* mutation is one of the most frequent primary mutations, but mutations in position D835 of the FLT3-tyrosine kinase domain (TKD) (9% of FLT3 mutations) are observed in 1328 samples (Supplementary Table S10), which we analyse in more detail below. The propensity of hotspot mutations to localize to protein interacting sites could alter the functions of proteins by affecting their interactions. Therefore, we analysed protein structures further at the atomistic level.

### The short loop similarity of a protein reveals functional complementing roles

**Figure 3.**
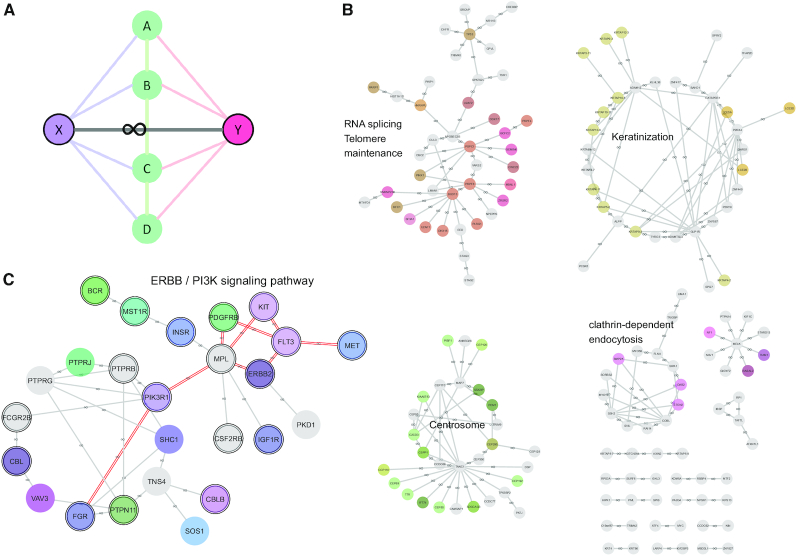
Schematic representation of short loop commonality and a landscape of short loop commonality among proteins with mutations in the AML PPIN. (**A**) ‘Short loop commonality’ is defined as the association of proteins having the same or similar short loop interactions, but not involving direct interaction of the proteins themselves. For example, X forms short loops with AB, BC and CD pairs and Y also forms short loops with AB, BC and CD but there is no direct interaction between X and Y. The short loop commonality pair of X and Y is annotated with a symbol of a loop (∞). (**B** and**C**) Short loop commonality, similarity of short loops between proteins, was calculated by comparing sets of protein interacting partner pairs for all proteins in the AML PPIN. In total 183 proteins (shown in light blue circles) account for 224 pairs of short loop commonality, which are annotated as line edges with a loop (∞) symbol. Functional enrichment of each cluster was measured by ClueGO (61) and nodes are coloured when proteins have enriched functional terms in the cluster. Detailed protein pairs and enriched functional terms are listed in Supplementary Tables S11 and S12. (B) Among the commonality pairs, a cluster consists of proteins involved in the PI3K pathway. This cluster can be divided into two subsets enriched with the RTK proteins (e.g. FLT3, KIT, ERBB2, PDGFRB and MET (right)) and their short loop interacting partners (e.g. PIK3R1, PTPRJ, PTPN11, CBL and CBLB (left)). Nodes with double border lines are druggable or potentially druggable proteins predicted by DGIdb (81) and edges are annotated in double red lines when a commonality pair has common drug interactions. The whole short loop commonality networks in the AML PPIN are available in a Cytoscape file format (ShortLoop_Commonality_leukaemia.cys) uploaded in (https://github.com/suns-chung/ShortLoopCommonality/).

Short loop network motif profiling was applied to the PPIN containing proteins mutated in AML and topological and functional analyses were carried out on the short loops identified that contain mutated proteins. Among the enriched short loops, we found some proteins do not directly interact but participate in similar short loop interactions, that is, they engage in PPIs with the same proteins which in turn interact with each other (example shown in Figure 3A). We defined the term ‘short loop commonality’ (commonality: ‘The state of sharing features or attributes’: https://en.oxforddictionaries.com/definition/commonality) to describe such protein relationships. To reduce bias caused by proteins having only a few short loop interactions, we therefore defined short loop commonality proteins based on two criteria: (i) at least three short loops are shared between the proteins in a commonality relationship and (ii) 95% of their short loops are in common.

**Figure 4.**
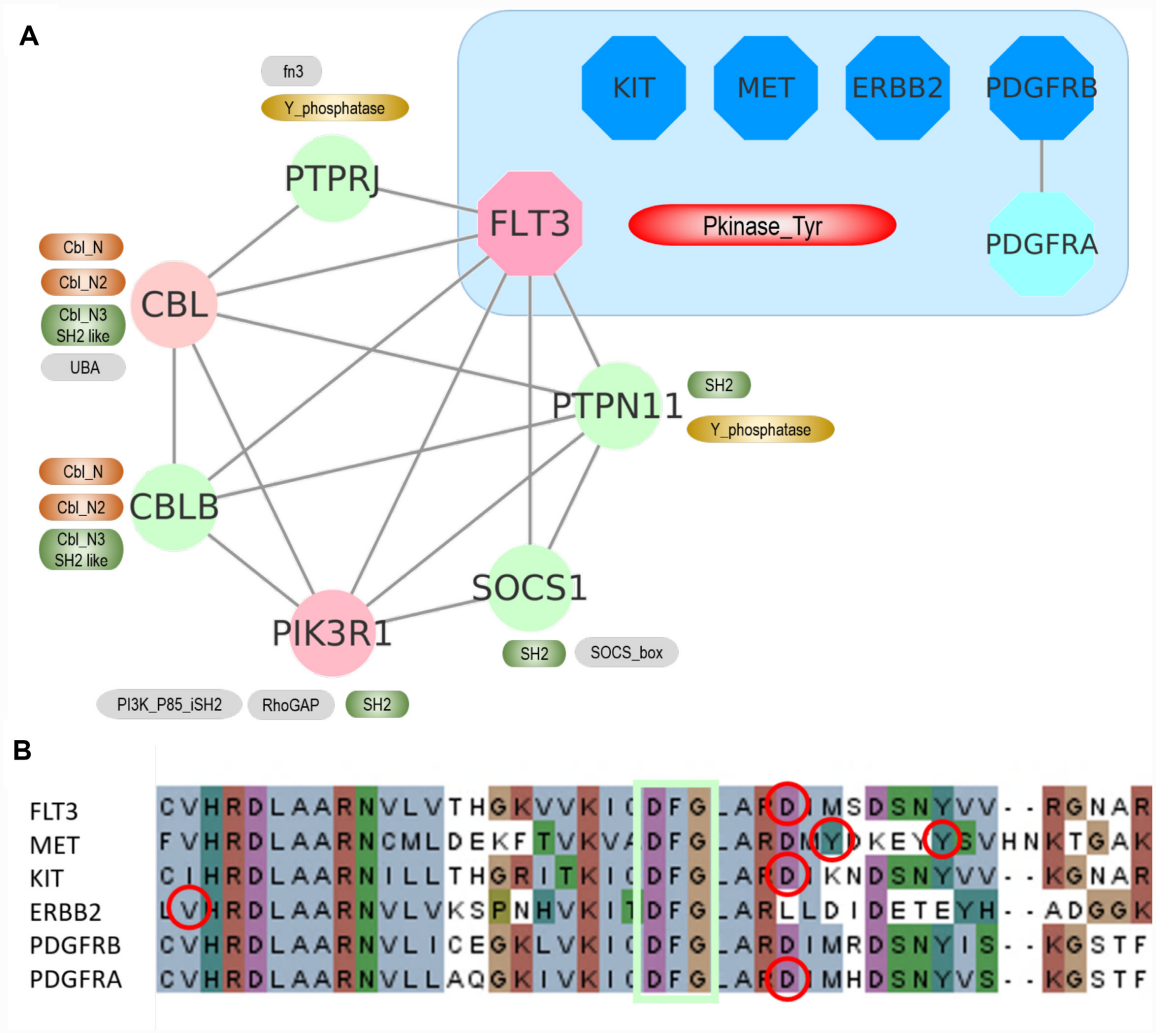
FLT3 short loop interactions and short loop commonality proteins. (**A**) The left side shows that FLT3 (pink octagon) has short loop protein–protein interactions with PTPN11, PTPRJ, SOCS1, PIK3R1, CBLB and CBL proteins, annotated as circles. Their protein–protein interactions are drawn as edges. Next to each protein, their functional domains are marked in round edged boxes. The right upper side in the blue area shows FLT3 short loop (length = 3) commonality proteins KIT, MET, ERBB2, PDGRB in blue octagons having the same short loop protein interactions as FLT3. (**B**) Amino acid sequences of FLT3 and its commonality proteins are aligned. The protein kinase domains of each protein were aligned by T-coffee (http://tcoffee.crg.cat/) and visualized by Jalview (http://www.jalview.org/). DFG motifs are boxed in light green and the hotspot mutations of each kinase are circled in red.

In the network containing proteins mutated in AML (total 2609 proteins with 14 119 PPIs), 183 proteins form 224 protein pairs that are in short loop commonality with each other (Figure 3B,C and Supplementary Table S11a) and there are six communities or clusters of the commonality pairs involving >5 proteins in each. The average number of neighbours in the AML commonality network (2.45) is higher than those of the other three leukaemias and simulated networks (*z*-score = 2.82) (Supplementary Table S11b). In addition, the degree of connectivity of the short loop commonality proteins in the PPIN (29.88 in AML PPIN) is higher than the average of the network (10.82) (Supplementary Table S7). This suggests that short loop commonality can be used as a measure of the interconnectivity of PPINs in addition to other topological properties of the network (Supplementary Table S7). Proteins in each cluster have enriched functions based on ClueGO analysis (61), such as RNA splicing, keratinization, centrosome organization and phosphatidylinositol 3-kinase (PI3K) signalling (Supplementary Tables S12 and S13), which may play a role as a functional unit or ‘module’. Among these clusters, a cluster of 25 proteins enriched in the PI3K pathway consists of two sub-clusters, one being the receptor tyrosine kinase (RTK) family such as FLT3, KIT, PDGFRB, ERBB2 and MET and the other with short loop interaction partners of these RTK proteins involving PIK3R1, PTPN11, PTPRJ, CBL and CBLB (Figures 3C and 4). These two sub-clusters are connected by a short loop commonality pair of MPL and PIK3R1 that have short loop PPIs with JAK2, SOCS1, SHC1 and PTPN11 (Supplementary Table S11a). These RTK commonality pair proteins can be targeted by common tyrosine kinase inhibitors such as Sorafenib (81) implying their co-functions in cellular processes (Figure 3C and Supplementary Table S14). Moreover, the short loop interactions related to these RTK proteins (Figure 4) are enriched with Src Homology 2 (SH2) domains that are present in five out of six proteins. Thus, such PPIs with predominant functional domains lead us to the hypothesis that the functions of the RTK family are shared or overlap in the underlying short loop protein–protein interactions and therefore functional commonality of frequently mutated proteins in cancers could be enriched in such short loops.

### FLT3 short loop commonality contains mutation hotspots in cancers

**Figure 5.**
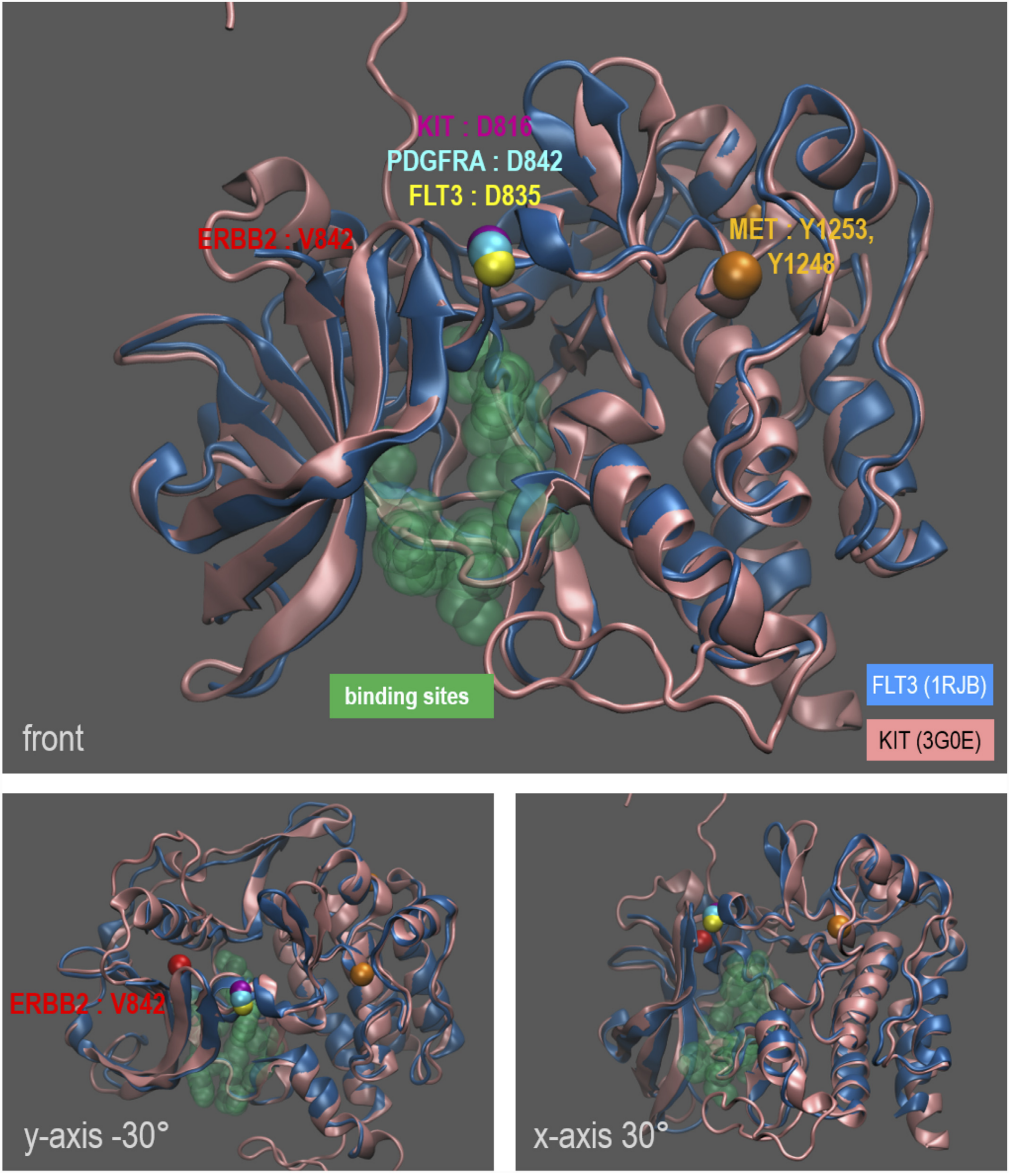
A zoomed-in view of the structural alignment of FLT3 and KIT proteins with mutation hotspots of FLT3 short loop commonality proteins in 3D space. FLT3 (1RJB) and KIT(3G0E) kinase domain structure 3D superimposition. The mutation hotspots of other FLT3 short loop commonality proteins are shown in beads representation and mapped onto this superimposition (FLT3: yellow, KIT: magenta, PDGFRA: light blue, MET: orange, ERBB2: red). To show the vicinity of mutation hotspots and the known small molecule binding site, this site is annotated on the original KIT structure and visualized as a green surface (top). Views from different angles are shown, rotating –30° in the *y*-axis (bottom left) and 30° in the *x*-axis (bottom right).

Mutations in kinase proteins have been extensively studied to understand their roles in cancer mechanisms (82,83) and mutation hotspots in these proteins are observed in various cancers (84,85). We hypothesize that when mutated members of the RTK proteins are in a short loop commonality relationship, mutations will be in mutation hotspots in multiple cancers. By investigating these RTK proteins using mutation data from the COSMIC database, we found that FLT3 and its short loop commonality RTK proteins have frequently mutated amino acid positions or mutation ‘hotspots’ in their kinase domain in various cancers (Supplementary Table S15) and in particular FLT3 is one of the most mutated genes in AML patients with a hotspot mutation (Figure 2 and Supplementary Table S10). By analysing the ratio of the occurrence of the mutation hotspot to the total number of mutations in the corresponding RTK proteins in all cancer types (defined as Mutation Hotspot Ratio Density (MHRD), ($MHRD\ = \frac{{{\rm{number\ of\ mutations\ on\ mutation\ hotspot\ in\ all\ cancer\ types}}}}{{{\rm{number\ of\ mutations\ in\ the\ protein\ from\ all\ cancer\ types}}}}$)), we observe this number to vary between 3 and 30%. All the ratios are statistically significant (*P*-value < 0.05, *z*-score > 3), against a null uniform distribution of mutations in the protein sequences (Supplementary Table S15). Since the effect of the mutational hotspot will be dependent on its spatial position in the protein structure, we analysed the specific location of mutation hotspots in the kinase domains of FLT3, and its short loop commonality proteins, in 3D structural space as well as in the linear amino acid sequences (Figures 4B and 5). Figure 4B shows that the mutation hotspots are closely aligned and located near the amino acid residues, Asp-Phe-Gly (DFG) motif typical of protein kinases, known as a ‘gatekeeper’ of protein kinase activities (86). They are in the activation loop of the kinase domain that is located near ligand or small molecule binding sites (87). Because of this 3D spatial closeness between mutation hotspots and functional sites, such mutations could interfere with protein–ligand interactions.

### Gene dependency screens corroborate topological ‘short loop commonality’

**Figure 6.**
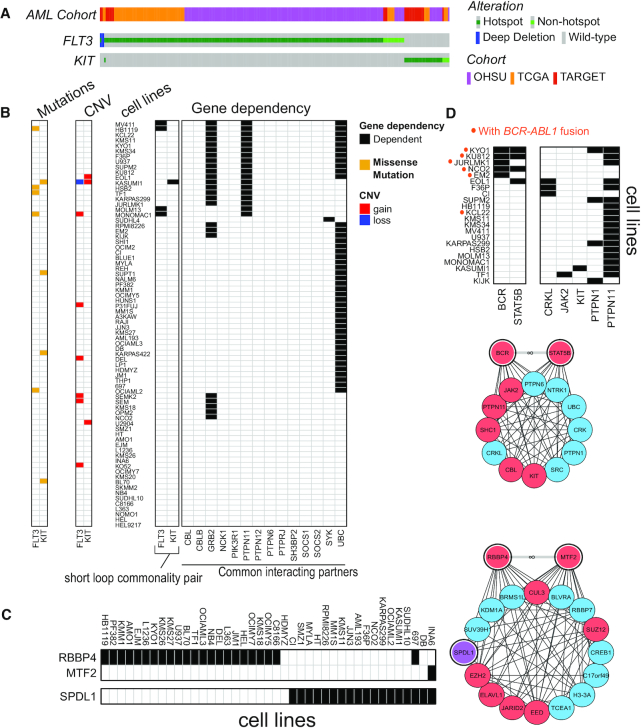
Gene dependency patterns reflect topological short loop commonality. (**A**) Mutations in the FLT3 and KIT genes in three AML clinical cohorts: OHSU (*n* = 622) (83), TCGA (*n* = 200) (65) and TARGET (paediatric AML *n* = 295) (84). Obtained from cBioportal (accessed 4 May 2020). (**B**) Gene dependency of FLT3, KIT and their common interacting partners (arranged on the horizontal axis) across haematopoietic cancer cell lines (vertical axis). Here CRISPR gene knockdown screening data from the AVANA project were obtained from depmap and visualized. Genes with a dependency score < –1 in cell lines were taken as ‘dependent’ genes. The SNV and CNV profiles of FLT3 and KIT in these cells are shown on the right. (**C** and **D**) Gene dependency of additional examples of short loop commonality and their common interacting partners. Data are visualized as in panel (B). Here only cell lines dependent on at least one of the genes of interest are shown. Genes that are not dependent in any of the cell lines are also omitted for simplicity. The interaction networks involving these molecules are shown next to the dependency plots. Proteins are annotated as nodes (red ones in leukaemias and blue ones in UniPPIN except for SPDL1 (purple)) and their interactions as black edges. Commonality pairs are in grey edges with ∞ signs and their nodes are highlighted in double lines.

The analysis above mainly focuses on annotating FLT3, KIT and their common interactors from the protein structural perspective. Here we determined whether the FLT3-KIT short loop commonality, as well as other cases we identify in the leukaemia-specific networks, is supported by cellular functional data. Specifically, we seek to determine whether there are patterns of protein dependency within a short loop commonality. We reason that in the case of FLT3 and KIT, the fact that their mutational hotspots overlap in 3D space and that they share common interacting partners is evidenced that cells may be dependent on either FLT3 or KIT but not both, since constitutive activation of FLT3 and KIT would have invoked interactions of the same set of proteins and therefore the same functional pathways. We observe that in clinical cohorts FLT3 and KIT hotspot mutations are mutually exclusive (one-sided Fisher exact test *P* = 0.002) (Figure 6A). We next leverage large-scale CRISPR–Cas9 screens of hematopoietic cancer cell lines with known mutations, where genes are deleted one at a time and cells are profiled for their viability, from which the dependency of the cell line on the gene is determined (see ‘Materials and Methods’ section). In these screens, cells are dependent on either FLT3 or KIT, but not both (Figure 5B). By overlaying the genomic profile onto these data, dependency on FLT3 and KIT are exclusive to those cell lines harbouring a FLT3/KIT hotspot mutation and/or copy number gain, suggesting oncogenic addiction (88) to either FLT3 or KIT (89,90). Surveying common interacting partners of the two proteins, we further observe that cell lines dependent on FLT3 or KIT are also dependent on PTPN11 and GRB2 (Figure 6B), suggesting that these two are common downstream signalling proteins of FLT3 and KIT. These data suggest that PTPN11 and GRB2 are potential drug targets in cells with activated FLT3 or KIT (91–93) and *in vitro* experiments using PTPN11 or GRB2 inhibitors could be designed to test such hypothesis.

We further ask whether other short loop commonality identified in our leukaemia networks exhibit the same pattern in the gene dependency screening data. By examining statistical independence between pairwise dependency profiles, we identify additional pairs of proteins that share short loop commonality and exhibit either mutual exclusivity (as does FLT3 and KIT) or co-occurrence of dependency in haematopoietic cancer cell lines. These protein pairs and their common interacting partners have known functions in common, including e.g. RBBP4 and MTF2 (Figure 6C), both of which are involved in chromatin remodelling and assembly complexes. Interestingly, dependencies of RBBP4 and its interacting partner SPDL1 are also mutually exclusive; this is consistent with the fact that the MuvB complex, of which RBBP4 is a member, represses *SPDL1* gene expression (94). Thus, RBBP4 and SPDL1 knockouts confer the same effect with respect to *SPDL1* expression level. We also observe cases where cells are dependent on both proteins sharing short loop commonality. For example, cells harbouring the *BCR-ABL1* gene fusion are dependent on BCR as well as STAT5B (Figure 6D), in line with previous studies (95–97). In general, we were able to identify more cases of mutual exclusivity and co-occurrence of gene dependencies between commonality pairs in the leukaemia networks (13 pairs of gene dependency mutual exclusivity and 3 co-occurrence), in comparison to a ‘control set’ where one member of each commonality pair was replaced with another protein which do not record any leukaemia mutations but still share common interacting partners (4 pairs of mutual exclusivity and 0 co-occurrence). The complete lists are in Supplementary Table S4. Altogether, this analysis suggests that short loop commonality identified as a topological feature from interaction networks also harbours functional significance that is supported by gene dependency screening data. These functional dependencies also bear therapeutic significance: for instance, in cases where the mutated protein is not druggable, our analysis suggests proteins sharing short loop commonality, or their common interacting partners, could be alternative drug targets, as they are involved in the same set of PPIs and therefore participate in the same functional pathways.

## DISCUSSION

Proteins and their interactions form the complicated intracellular ‘machinery’ that carry out and regulate the functions of cells in our body. Diseases are outcomes of malfunctions in this machinery caused in part by genetic abnormalities that change the functions of the encoded proteins. Cancer pathologies are the epitome example showing the complexity of disease mechanisms affected, which disrupt diverse cellular functions. Thus, building a comprehensive protein–protein interaction network of a cell is an important step towards an understanding of the complexity of molecular disease mechanisms, which could lead to the development of more targeted therapies. Several databases provide amalgamated information on protein interaction data from multiple sources. However, each of these represent distinct subsets of the protein interaction landscape. Data were collected using different experimental methods, stored in multiple locations and in disparate data formats. These pose challenges to amalgamate them to form a more complete human proteome map. The UniPPIN presented here, a unified human protein–protein interaction network, integrates multiple resources of human PPIN covering 19 370 proteins with 385 370 interactions. This extends the data in a recently curated large-scale human protein interaction map integrating high-confidence mass spectrometry experiments, which covers >7700 proteins, and >56 000 unique interactions (98). The UniPPIN proteome map is designed to deepen our understanding of human proteomes, as well as the functional relationship between genotypes and phenotypes, especially to extend protein coverage for diseases such as AML. However, the extent of a large PPIN poses challenges in extracting data of specific PPIs that are relevant to a given disease. To acquire such information, we applied our short loop network motif profiling method (38) to the amalgamated UniPPIN, annotated with data on proteins mutated in four different leukaemias. We also develop a new concept—‘short loop commonality’—and propose that protein ‘modules’ that form short loops with different, related proteins may select for a class of hotspot mutations that affect protein–protein interactions.

In our previous study (38), short loops (cyclic protein interactions) were shown to contain topological and biological information about PPINs as they form a core of resilient interconnections with enriched biological functions. This analysis method demonstrated its utility by retrieving specific and unappreciated topological and biological properties of the networks. Here we used this approach to analyse PPINs containing mutated proteins in four different leukaemias. The high ratio of short loops in the AML-related PPIN shows that mutations reported in AML are more interconnected in the underlying proteome than those in the other leukaemia PPINs; our analyses also indicate that mutated proteins in AML play roles in a broad range of biological processes (Figure 1). In addition, proteins frequently found in AML short loops overlap with resilient loops of a reliable human PPIN from our previous study (38). Resilient loops compose a highly connected core of PPIs since they are preserved after randomization of the interactions based on Markov Chain Graph Dynamics. Also, they contain proteins involved in essential cellular functions (38). This indicates that some AML short loops are resilient, comprised of proteins involved in essential functions including transcription, RNA processing, hnRNA splicing and translation (Supplementary Table S8).

Here, we extend the short loop profiling approach and present a novel concept called ‘short loop commonality’ to analyse indirectly connected proteins having the same short loop interactions. The method can identify communities of proteins or ‘modules’ related to particular functions. We observe co-localization of hotspot mutations in the FLT3 kinase and other RTK commonality members, suggesting that these mutations interfere with protein–ligand interactions. Additionally, these short loop commonality proteins show mutual exclusivity or dependency in gene knockout screening data. Dependency means that cell survival is dependent on an interacting partner of a mutated protein in a short loop commonality, implying that the second protein could be a relevant drug target if the mutated protein is not. Exclusivity is inferred when cells are mutated in only one but not both members of a short loop commonality pair. These cases highlight the potential of the short loop commonality topological measure in discovering functional redundancy or synthetic lethality of proteins (99).

Network biology has improved our understanding of biological systems related to diseases by implementing models based on topological properties of intracellular networks (34,100,101). The aim has been to identify geno-/phenotypic associations in diseases and ultimately to develop novel translational approaches (100,101). Also, recent efforts on human protein interactomes mapped with disease associated proteins have been used to predict how protein abnormalities can affect protein complexes (8,53). However, mapping mutation information onto PPINs is still hampered by proteome coverage and the sparsity of information about the sub-networks affected by mutations. The concept of short loop commonality is a promising method for analysing PPINs, identifying functional dependency of proteins and finding underlying proteins which affect disease-related mechanisms. It supports a paradigm shift in the drug discovery approach from a one-mutated target-one drug model to a multiple-target strategy for the local PPIN of a single mutated protein (102). Many mutated proteins are not suitable currently for drug discovery and we propose that our approach may be useful in identifying functional protein modules affected by short loop commonality mutations that are potential new drug targets. In addition, it could also help in identifying repurposed drug candidates (103), for instance, if one drug targets a protein within a short loop commonality, the drug might be used to treat malignancies related to the commonality pair, that is, where the interacting protein of the drug target is mutated. Examples are common drugs targeting short loop commonality pairs in leukaemias, such as tyrosine kinase inhibitors including Sorafenib (81) and PI3K inhibitors including Pictilisib (104) (Supplementary Table S14 and Figure 3C). Our analysis using CRISPR–Cas9 screening data suggests short-loop commonality proteins do harbour functional dependencies, which could be exploited in cases where knowledge on ‘druggability’ of proteins is incomplete, or where targeting specific proteins proves ineffective. This could provide twofold benefits by tailoring druggable targets to a robust ‘module’ complex, and by prioritizing interacting partners which remain unperturbed by mutations as potential targets for therapeutics. These targets would have been overlooked solely using mutation data. For example, PTPN11, a protein interaction partner of FLT3 and KIT short loop commonality pair, could be a therapeutic target for AML patients with activating FLT3 or KIT mutations (92,93,105).

With the present study, we demonstrate that short loop network profiling can be used to analyse genome-wide data of mutations in cancers. This approach highlights the underlying functional consensus of short-range protein interactions in PPINs. Furthermore, proteins that do not necessarily interact share short loop interactions that could be used to identify essential PPI modules that affect important cellular functions. Mutations in components of these short loops contribute to cancer and we observe that the short-loop components harbour mutational hotspots. This knowledge can be exploited in the design of functional experiments: by identifying components of short loops, this helps in identifying proteins with measurable phenotypic readouts that could be harnessed to study the effect of mutations, especially if such mutations fall in proteins which are difficult to be probed experimentally. A map of co-dependency of proteins in short loops could also be helpful in prioritizing interacting partners as new therapeutic targets in cases where a mutated protein is difficult to target pharmacologically. These associations will ultimately stimulate the investigation of new targets in protein modules of the commonality for drug discovery and drug repurposing in a disease such as AML where no targeted therapies have proved effective (47). The terms ‘short loop network motif’ and ‘short loop commonality’ defined here can be used in the future when new experimental data of time- and scale-dependent personalized PPINs are available that enable us to quantify the evolution of network changes in many diseases.

## Supplementary Material

lqab010_Supplemental_Files

## Notes

Present address: Sun Sook Chung, Institute for Protein Innovation, Boston, MA, 02115, USA.

Present address: Anna Laddach, Development and Homeostasis of the Nervous System Laboratory, The Francis Crick Institute, London, NW1 1AT, UK.

Present address: N. Shaun B. Thomas, Professor Emeritus of Blood Cell Biology, King’s College London, UK.
